# Identifying epithelial borders in cholesteatoma surgery using narrow band imaging

**DOI:** 10.1007/s00405-021-07045-4

**Published:** 2021-08-22

**Authors:** Adrianus H. A. Baazil, Maura C. Eggink, Maarten J. F. De Wolf, Fenna A. Ebbens, Frederik G. Dikkers, Erik van Spronsen

**Affiliations:** Department of Otorhinolaryngology, Amsterdam UMC, University of Amsterdam, Meibergdreef 9, 1105 AZ Amsterdam, The Netherlands

**Keywords:** Narrow band imaging, Cholesteatoma surgery, Epithelial border, Residual disease

## Abstract

**Purpose:**

To quantify changes in the perceived epithelial border with narrow band imaging (NBI) and white light imaging (WLI) during cholesteatoma surgery and to objectify possible benefits of NBI in otology.

**Methods:**

Perioperative digital endoscopic images were captured during combined approach tympanoplasty at our tertiary referral center using WLI and NBI (415 nm and 540 nm wavelengths). Sixteen otologic surgeon defined the epithelial borders within 16 identical WLI and NBI photos. Pixels of these selections were calculated to analyze the quantitative difference between WLI and NBI. A questionnaire also analyzed the qualitative differences.

**Results:**

Sixteen otologic surgeons participated in the study. Stratified per photo, only two photos yielded a significant difference: less pixels were selected with NBI than WLI. A Bland–Altman plot showed no systemic error. Stratified per otologist, four participants selected significantly more pixels with WLI than with NBI. Overall, no significant difference between selected pixels was found. Sub-analyses of surgeons with more than 5 years of experience yielded no additional findings. Despite these results, 60% believed NBI could be advantageous in defining epithelial borders, of which 83% believed NBI could reduce the risk of residual disease.

**Conclusion:**

There was no objective difference in the identification of epithelial borders with NBI compared to WLI in cholesteatoma surgery. Therefore, we do not expect the use of NBI to evidently decrease the risk of residual cholesteatoma. However, subjective assessment does suggest a possible benefit of lighting techniques in otology.

**Level of evidence:**

3.

**Supplementary Information:**

The online version contains supplementary material available at 10.1007/s00405-021-07045-4.

## Introduction

Narrow band imaging (NBI) is a relatively new modality in the field of otorhinolaryngology. It remains under investigation how useful it will be in specifically otology. To our knowledge, only two studies described the possibilities of NBI in otology [1, 2]. In the first study, the authors qualitatively describe the perioperative images captured with NBI. In the second study, the authors captured images of normal and pathologic tympanic membranes and compared the difference in contrast. Both studies concluded that NBI could be of added value, but they lacked objectivation and quantification of the proclaimed advantages.

One of the advantages hypothesized was the improved accuracy in detection of epithelial borders in cholesteatoma surgery [1]. Narrow band light exists of two wavelengths that penetrate the surface of the tissue (415 nm and 540 nm) and is mainly absorbed by hemoglobin in blood vessels [3]. It has been proven to be a useful endoscopic tool in the diagnosis of benign and malignant mucosal pathology, such as recurrent respiratory papillomatosis and squamous cell carcinoma [4–9]. Theoretically, this characteristic could be utilized to differentiate skin, being an avascular structure, from other vascularized tissues [1]. NBI could, therefore, be beneficial for recognition and removal of epithelium in cholesteatoma surgery. Endoscopes have also been deemed useful in cholesteatoma surgery, due to improved middle ear exposure and movability [10]. Perhaps, a synergetic effect will be achieved when these modalities are combined.

In this study, we aim to quantify changes of the perceived border between epithelium and other tissue with NBI and white light imaging (WLI). We hypothesize that the epithelial border will be perceived differently in comparison to WLI and that NBI will accentuate suspicious lesions which would be missed with WLI.

## Methods

### Photos for evaluation

**Fig. 1:**
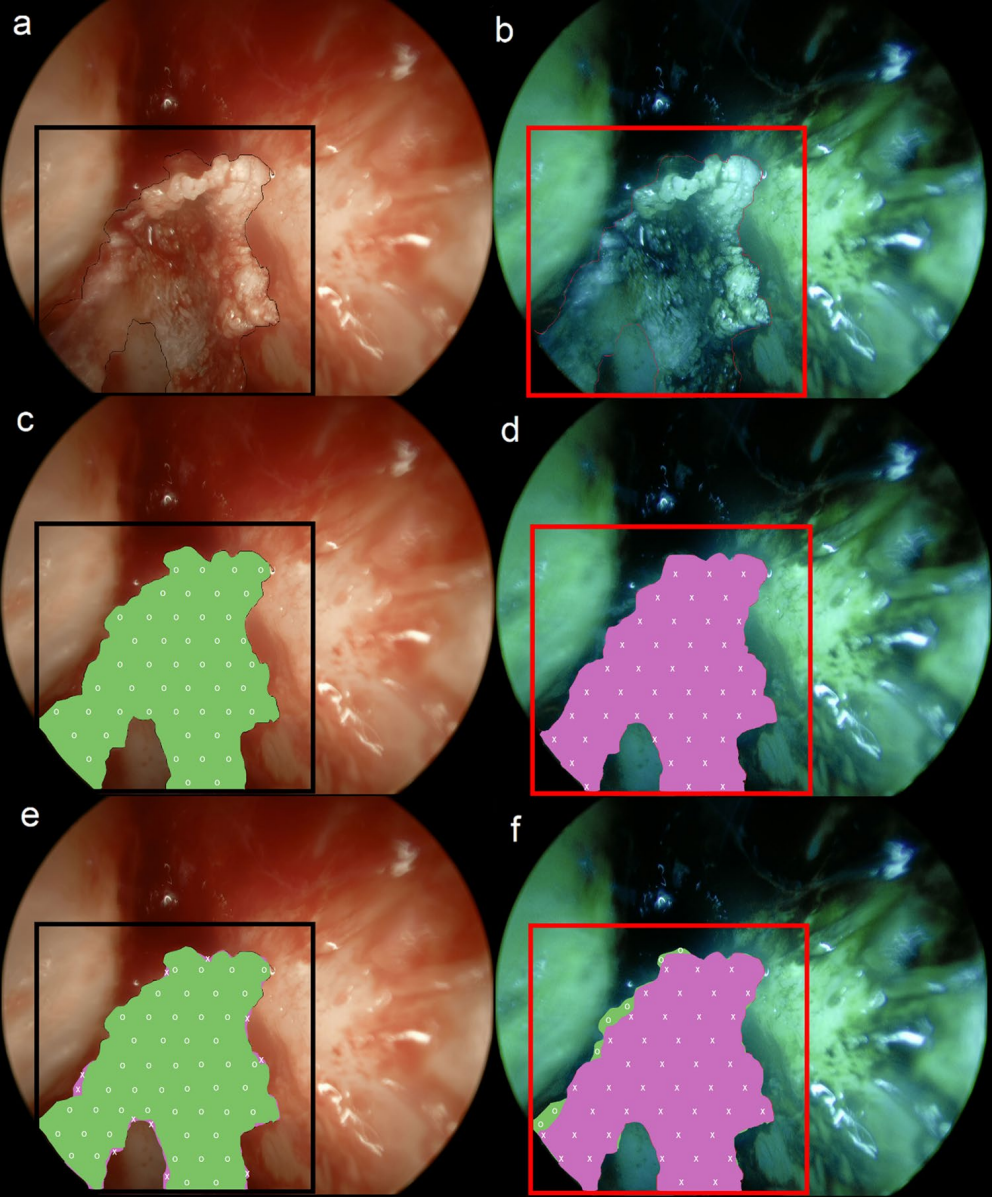
Example of white light (WLI) and narrow band imaging (NBI) photos of the left ear during combined approach tympanoplasty. **a**, **b** shows perioperative WLI and NBI photos of the left antrum during cholesteatoma surgery, with the external ear canal on the left and the tegmen tympani on the right. The box represents the framework in which the epithelial border is drawn by the participant. In **c**, **d**, the drawn borders are used to make the pixel selection and add a green (o) or purple (x) colored layer with Photoshop. The layers are projected over one another in (**e**, **f**)

Perioperative photos were taken during cholesteatoma surgery between July and August 2020. After a retroauricular surgical approach was performed, with subsequent opening of the mastoid cavity and the cholesteatoma sac, keratin was removed from the sac. The epithelial border was identified. The surgical field was maximally cleared of blood by rinsing with water and local application of noradrenaline (1:1000). An Olympus 0-degree rigid laryngoscope of 5 mm in diameter was used, using a 4 K Olympus NBI system (415 nm and 540 nm wavelengths). After applying the auto-focus function to ensure sharp epithelial borders, digital endoscopic images were captured. Two consecutive photos were taken, one with WLI (Fig. 1a) and one with NBI (Fig. 1b).

As photos were two dimensional, the center of photos was in focus and the periphery was blurred. A framework was drawn in all photos, with Microsoft Paint, containing only the focused part of the photo, in which the epithelial border was to be defined. A short description of the image was supplied to aid orientation of the surgical situation.

### Participants

Otologic surgeons in the Netherlands were approached to participate in this study. Their otologic experience was noted, according to registration by the Dutch ENT society. The images were sent to the participants digitally and evaluated individually. The participants had no prior training in using NBI and declared to have no conflict of interest in NBI, neither personal nor commercial. Epithelial borders were defined and drawn with the smallest width pencil tool in Microsoft Paint, to ensure precision. In the WLI photos, a black color was used (Fig. 1a), while in the NBI photos a red color was applied (Fig. 1b).

### Data analysis

Photos were divided into two groups. Group 1 consisted of ten WLI and their corresponding NBI photos. The corresponding WLI and NBI photos were subsequently evaluated. Participants were allowed to switch between photos before defining the border. This was done to evaluate if the WLI photo would influence the location of the perceived border with NBI. As participants were not trained in the use of NBI, this would allow them to get accustomed to the visual effect of NBI. In group 2, six WLI and their corresponding NBI photos were presented and evaluated in a random order. In this group, it was not permitted to switch between photos before defining the border. Otologic surgeons were asked to analyze all photos in both groups.

With the Magnetic Lasso tool from Adobe Photoshop (version 13.0.1), borders were selected and the perceived area of epithelium encircled. Pixels within the defined area were quantified by the software. For all photos, the total number of pixels was identical. The selected pixels from the WLI photos were colored green (Fig. 1c) and the NBI-selection purple (Fig. 1d). As the WLI and NBI photos were identical, selected areas and overlap could be compared to one another. NBI selections were projected over the WLI selection, and vice versa (Fig. 1e, f). NBI pixels which overlapped with WLI pixels were quantified by the software and expressed as a percentage of the total amount of NBI pixels.

### Questionnaire

Participants filled out a questionnaire including three questions. First, participants were asked if NBI was advantageous over WLI in defining the epithelial border. Second, the ability of NBI to reduce residual disease was questioned. If so, the amount of money the otologists were prepared to pay for the NBI system had to be selected (A: €1000, B: €5.000, C: €10.000, D: €50.000 and E: €100.000).

### Statistical analysis

*Z* values for skewness and kurtosis were evaluated to analyze normality of the data (data were considered normally distributed if − 1.96 < *z* < 1.96). Normally distributed data were expressed as mean with its standard deviation. Non-parametric data were expressed as median with its interquartile range. Differences between WLI and NBI selections were assessed with paired samples *t* test and Wilcoxon signed-rank test. A *p* value less than 0.05 was considered statistically significant. A Bland–Altman plot was made to evaluate a possible systemic error in differences. Linear regression analysis was done to compare participant evaluations.

### Ethics

The authors assert that all procedures contributing to this work comply with the ethical standards of the relevant national and institutional guidelines on human experimentation and with the Helsinki Declaration of 1975, as revised in 2008. Approval from the institutional review board was not necessary as photos were anonymous and no patients were investigated.

## Results

All photos were acquired in ten different operations in which a combined approach tympanoplasty was performed, both primary and revision cholesteatoma surgery. Sixteen photos of the highest quality were selected, corresponding to 16 WLI photos and 16 identical NBI photos.

Sixteen otologic surgeons participated in the study. All otologists were employed at regional hospitals across the country. Of these otologists, 11 had more than 5 years of experience.

### Evaluation by participants stratified per photo

In 2 of the 16 photos, both from group 1, a significant pixel difference was found between the WLI and NBI selection (paired samples *t* test, *p* < 0.05). For these two photos, NBI resulted in a lower number of selected pixels. For the other 14 photos, there was no significant difference. In appendix A (Online Supplementary material), the selected pixels per WLI and NBI photo are shown with corresponding *p* values.

In total, a median of 93.5% (IQR 10.4%) NBI pixels overlapped with the WLI selection. In group 1, this median was 93.4% (IQR 10.6%) and in group 2, it was 94.1% (IQR 9.5%). The difference between both groups was not statistically significant (Wilcoxon signed-rank test, *p* > 0.05).

**Fig. 2:**
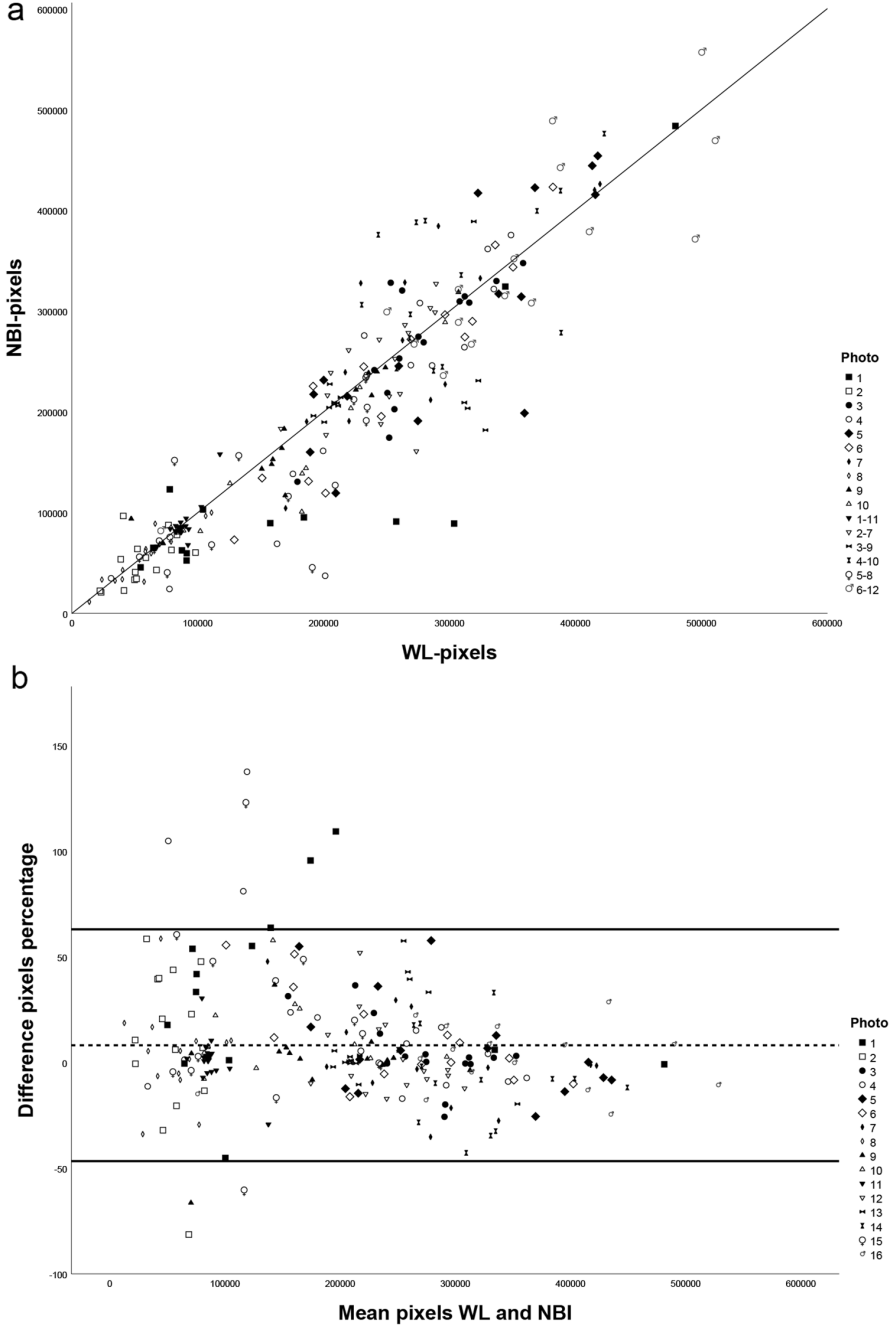
**a** Scatterplot of white light (WLI)- and narrow band imaging (NBI)-selected pixels for all photos showing limited differences between the two modalities. Photos 1–10 were evaluated in order (group 1). Photos 11–16 were evaluated in random order (group 2). **b** Bland–Altman plot of the mean and difference between white light (WLI) and narrow band imaging (NBI) pixel selection illustrating no systematic error. Difference is plotted on the y-axis as a percentage of the mean. The dotted line represents the mean difference and the black lines correspond with ± 2 standard deviations. For photo 1 and 10, a mainly positive difference can be seen that leads to a significant difference: WLI > NBI. For all other photos, differences are distributed randomly

Figure 2a shows a scatterplot in which the WLI and NBI pixels of individual photos are plotted. Comparison with the drawn *y* = *x* line shows that there is a small difference for most photos between the WLI and NBI selection. A Bland–Altman plot (Fig. 2b) shows no systematic error.

### Evaluation by participants stratified per otologic surgeon

**Fig. 3:**
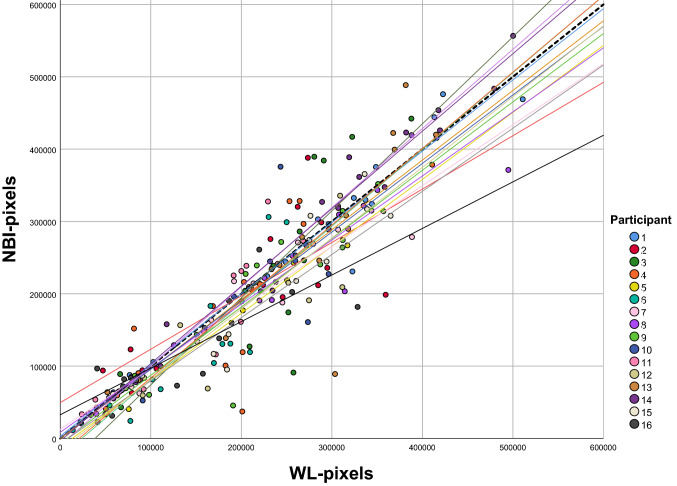
A scatterplot of white light (WLI) and narrow band imaging (NBI) pixels of individual participants, with corresponding linear regression lines, demonstrates the small difference between modalities. For 11 participants, the slope was smaller than 1, meaning that for 1 pixel of WLI selection corresponded with less than 1 NBI pixel selected. For five participants, the slope was larger than 1. Slopes generally are close to 1, thus close to the *y* = *x* line (dotted line)

When stratifying per otologic surgeon, participant 5, 12, 14 and 15 selected significantly more pixels with WLI than with NBI (paired samples *t* test, *p* < 0.05). A linear regression line was plotted for all participants and Fig. 3 illustrates most participants do not significantly differ from the *y* = *x* line. For 11 participants, the slope was smaller than 1, meaning that for 100 pixels of WLI selection less than 100 NBI pixels were selected. For 5 participants, the slope was larger than 1. Selections of participant 2, 3 and 16 were most divergent from the *y* = *x* line and resulted in slopes of, respectively, 0.738, 1.203 and 0.644. For these three participants, there was no significant difference in selected pixels between the two lighting modalities overall (paired samples *t* test, *p* > 0.05) as WLI pixel selections for some photos were larger than for NBI and vice versa.

Sub-analyses of the group of otologic surgeons with more than 5 years of experience, compared to surgeons with less seniority, also yielded no significant differences between NBI and WLI or number of pixels selected.

Some otologic surgeons were not able to submit all photos due to technical difficulties; therefore, not all image selections are complete [Table 1 and Appendix A (Online Supplementary material)]. Analyses were done with available images.

**Table 1:** Linear regression analysis and intra-participant *p* values of differences between white light (WLI)- and narrow band imaging (NBI)

Participant No	Available photos	Linear regression (β)	Intra-participant pixel difference (*p* value)
1	16	0.985	0.889
2	15	0.738	0.804
3	16	1.203	0.979
4	16	0.963	0.649
5	5	0.926	0.027*
6	16	1.062	0.137
7	16	0.842	0.099
8	16	0.886	0.075
9	15	0.945	0.115
10	15	0.948	0.548
11	16	1.095	0.461
12	11	0.873	0.031*
13	16	1.057	0.650
14	15	1.071	0.028*
15	15	0.978	0.030*
16	15	0.644	0.113

Wilcoxon signed-rank test was used. Statistically significant results are marked with an asterisk (*p* < 0.05)

### Questionnaires

Ten of 16 participants (63%) filled out the questionnaire. Six of them (60%) found NBI advantageous in defining the border of epithelium, of which five (83%) believed NBI could help reduce the risk for residual disease. These five participants were be prepared to invest €5.000 in an NBI system.

## Discussion

In this study, 2-dimensional photos were used to quantify and compare epithelial borders in cholesteatoma surgery by 16 participating otologic surgeons. This method allows for direct comparison between WLI and NBI in identical perioperative images to evaluate the potential added value of NBI in cholesteatoma surgery. By drawing a framework inside the photo, participants evaluated the area of the photo which was in focus and of highest quality. A potential drawback of this study is that it might not be a perfect representation of reality. First, as static photos were used, sight of depth and the possibility to dynamically visualize borders lack in this method. Second, as NBI colors blood black, hampering view of underlying tissues, it was purposely removed from the surgical field to allow for optimal visualization. It could be questioned whether this method is adequate and time efficient in a real-time surgical setting. Local application of noradrenaline could also influence the appearance of epithelium with NBI due to vasoconstriction. In this study, no correction was done for drawing error. Despite these minor limitations our study design, we consider it robust enough to draw solid conclusions.

Presently, two studies have been performed that describe the use of NBI in otology [1, 2]. Valdez et al. used a modified otoscope to obtain images of normal tympanic membranes, cholesteatoma and acute otitis media [2]. Zhang et al. looked at multiple ear pathologies, including cholesteatoma, with NBI [1]. Both state that NBI has advantages in identification and dissection of cholesteatoma [1, 2]. Neither study objectified nor quantified this presumed advantage. Our study provided no significant differences in the number of selected pixels with NBI in comparison to WLI for most photos when images were examined in both modalities separately and successively. Linear regression analyses illustrated that overall WLI-pixel selection was close to NBI-pixel selection. Furthermore, differences between WLI and NBI selections within participants were smaller than the variation between participants. This is reflected by the large standard deviation in comparison to the mean of WLI and NBI selections that was found in our study, suggesting a greater influence of participant on number of pixels selected than lighting modality. Also, for the few photos and participants in which a significant difference was found between NBI and WLI, more pixels were selected with WLI than with NBI. NBI would, therefore, result in a smaller perceived area of epithelium and theoretically less tissue resection. As it has been shown that more tissue resection leads to a lower chance of residual cholesteatoma [11, 12], this could be an evident disadvantage of NBI use. We demonstrated that over 93% of the NBI pixels overlapped with the already selected WLI pixels. As the remaining 7% of NBI pixels were mainly located adjacent to the WLI selections, it revealed no novel areas of epithelium. However, due to the method of the study, no perioperative biopsies could be taken of the areas of difference between WLI and NBI. We thus cannot state with certainty that no additional areas of disease were present. Overall, our findings weaken the previously suggested assumption that NBI could allow for increased accuracy in detection of epithelial borders. We, therefore, do not expect NBI to significantly decrease of the number of cholesteatoma residuals.

Despite the lack of uniform results, a majority of the participants considered NBI to be potentially beneficial in identifying epithelial borders, according to the questionnaires. They also believed NBI to be helpful in lowering cholesteatoma residuals. All of them were prepared to invest € 5.000 in an NBI system. This is in line with the possible benefit found in the literature. Therefore, it is possible that advantages of NBI do exist but were not demonstrated by our study. Lucidi et al. looked at the use of digital color renderings to enhance the spectral separation of the recorded broad visible spectrum to histologically confirm visual suspicion for cholesteatoma [13]. Digital color rendering was also used by Miwa et al. to grade normal tissue and cholesteatoma based on vascularization pattern and surface irregularity [14]. Digital color rendering is different from NBI as this is done by digital application of color filters during image processing. Lucidi et al. conclude that digital color rendering successfully enhances cholesteatoma tissue and results in high sensitivity and specificity rates [13]. A major drawback of this study was the lack of histological confirmation of visually unsuspicious tissues as WLI was used to declare a lesion unsuspicious of cholesteatoma. Miwa et al. conclude that scores for normal mucosa and cholesteatoma significantly differ [14]. For this study, histological confirmation lacked for suspected normal mucosa and cholesteatoma. Despite drawbacks of both studies, they also suggest that the use of different lighting modalities might be beneficial in cholesteatoma surgery.

The advantages of NBI in diagnosing mucosal pathology are based on better visualization of vascularization patterns and density [4–9]. The avascular structure of cholesteatoma could credibly be the reason no advantages were found in cholesteatoma surgery. Our findings do not suggest that NBI has no benefits in the entire field of otology. It would be interesting to evaluate changes in vascularization during treatment of granulomatous myringitis and evaluation of tympanic membrane vascularization after myringoplasty in relation to closing rates. More research should be done to evaluate these other potential benefits. As an NBI system is costly, a necessary next step would be to perform a cost–benefit analysis. Of course, these costs should be put in to perspective as the system can be of added value to the entire ENT department, especially laryngologists. Also, applicability in day-to-day practice has to be considered. As NBI is a built-in modality of light sources, it can be easily used during endoscopic surgery. At present, operation microscopes include multispectral functionality that enhance contrast, but do not emit true NBI wavelengths. NBI is not (yet) available on microscopic light sources. It would, therefore, be unpractical for perioperative microscopic use.

## Conclusion

Our data show that epithelial borders are not perceived significantly different with NBI to WLI. We, therefore, deem it unlikely that NBI will greatly contribute to complete removal of cholesteatoma. Subjective assessment of NBI does suggest that lighting modalities could still be beneficial in cholesteatoma surgery.

## Supplementary Information

Below is the link to the electronic supplementary material.

Supplementary file1 (DOCX 18 KB)
