# Supplementary material for: Identifying epithelial borders in cholesteatoma surgery using narrow band imaging
Source: Eur Arch Otorhinolaryngol. 2021 Aug 22;279(7):3347–54. doi: 10.1007/s00405-021-07045-4 (PMC9130169; doi:10.1007/s00405-021-07045-4)
Supplement: Supplementary file 1 — Supplementary file1 (DOCX 18 KB) [file 405_2021_7045_MOESM1_ESM.docx]

**Appendix A. Selected pixels per photo and modality with the inter-modality *p*-values and percentage of narrow band imaging (NBI)-pixels overlapping with white light imaging (WLI)-pixels.**

For normally distributed data mean with standard deviation (SD) is given and a paired t-test was done. For non-parametric data median with interquartile range (IQR) is given and a Wilcoxon signed-rank test was done. For photo 1 and 10 (group 1) a significant difference between the WLI- and NBI-selection was found. Their *p*-values are marked with an asterisk (*p*<0.05). No significant differences between both lighting modalities were found for the photos in group 2. A median of 93.5% (IQR 10.4%) of NBI-pixels overlapped with the WLI-selection.

| Photo No. | Modality | Available photos | Mean (SD) or Median (IQR) pixels in selection | Inter-modality difference of pixels (*p-*value) | NBI-pixels overlapping with WLI-selection (%) |
| --- | --- | --- | --- | --- | --- |
| Photos in order (group 1) | | | | | |
| 1 | WLI | 15 | 91 164 (172 231) | 0.015* |  |
|  | NBI | 15 | 89 095 (40 569) |  | 89.8 |
| 2 | WLI | 15 | 55 524 (21 848) | 0.511 |  |
|  | NBI | 15 | 51 715 (23 675) |  | 81.7 |
| 3 | WLI | 15 | 275 931 (44 450) | 0.445 |  |
|  | NBI | 15 | 268 118 (63 997) |  | 92.1 |
| 4 | WLI | 14 | 231 228 (96 375) | 0.097 |  |
|  | NBI | 14 | 204 521 (125 336) |  | 86.6 |
| 5 | WLI | 15 | 302 395 (86 349) | 0.499 |  |
|  | NBI | 15 | 290 848 (113 931) |  | 91.0 |
| 6 | WLI | 14 | 257 333 (78 248) | 0.154 |  |
|  | NBI | 14 | 241 953 (102 078) |  | 86.9 |
| 7 | WLI | 13 | 275 462 (77 120) | 0.728 |  |
|  | NBI | 13 | 281 019 (97 969) |  | 89.5 |
| 8 | WLI | 14 | 58 634 (27 868) | 0.556 |  |
|  | NBI | 14 | 56 835 (26 981) |  | 91.1 |
| 9 | WLI | 14 | 185 050 (70 348) | 0.623 |  |
|  | NBI | 14 | 182 169 (68 112) |  | 93.3 |
| 10 | WLI | 12 | 155 395 (71 226) | 0.027* |  |
|  | NBI | 13 | 136 666 (68 451) |  | 93.8 |
| Photos in random order (group 2) | | | | | |
| 11 | WLI | 16 | 88 070 (10 874) | 0.642 |  |
|  | NBI | 16 | 83 757 (8 775) |  | 92.8 |
| 12 | WLI | 16 | 245 134 (36 141) | 0.785 |  |
|  | NBI | 16 | 242 237 (50 295) |  | 91.2 |
| 13 | WLI | 16 | 210 964 (109 198) | 0.112 |  |
|  | NBI | 15 | 208 548 (10 860) |  | 93.0 |
| 14 | WLI | 16 | 305 836 (60 905) | 0.157 |  |
|  | NBI | 14 | 333 302 (75 135) |  | 82.8 |
| 15 | WLI | 16 | 152 182 (154 801) | 0.109 |  |
|  | NBI | 16 | 128 295 (70 505) |  | 90.6 |
| 16 | WLI | 16 | 347 766 (108 875) | 0.596 |  |
|  | NBI | 16 | 340 103 (113 468) |  | 90.7 |
